# Monitored hygrothermal dataset of a high-altitude Dolomite refuge: wall and indoor climate at 2870 m a.s.l.

**DOI:** 10.1038/s41597-025-06441-3

**Published:** 2026-01-07

**Authors:** Simone Panico, Alessandro Zandonai, Eleonora Leonardi, Marco Larcher, David Cennamo, Daniel Herrera-Avellanosa, Alexandra Troi

**Affiliations:** 1 Institute for Renewable Energies, Eurac Research, Viale Druso 1, 39100 Bolzano, Italy; 2 Institute for Alpine Environment, Eurac Research, Viale Druso 1, 39100 Bolzano, Italy

**Keywords:** Civil engineering, Environmental impact, Structural materials

## Abstract

A five-year hygrothermal dataset (23 September 2020 to 8 August 2025) was compiled from Rifugio Boé, a 1905 stone mountain refuge situated at 2870 m a.s.l. in the Dolomites. The dataset includes hourly recordings of outdoor climate parameters (temperature, relative humidity, global tilted irradiance, and driving rain), indoor conditions across six functionally distinct rooms, and temperature–humidity profiles at three depths within the retrofitted south-east wall. The monitoring system was engineered for robust, long-term operation under harsh alpine conditions. Data are provided in CSV format, accompanied by comprehensive sensor metadata. The dataset supports: (i) validation of hygrothermal simulation models under extreme alpine conditions; (ii) evaluation of the long-term performance and durability of internal insulation within historic masonry; and (iii) benchmarking of moisture-related risk in high-altitude heritage structures. Moreover, the dataset offers a rare opportunity to examine hygrothermal responses in high-elevation-built heritage, a research domain where long-term, high-frequency data remain scarce.

## Background & Summary

Numerical simulations are fundamental in architectural and engineering practice, though their accuracy greatly depends on input parameters and boundary conditions^1,2^. This issue becomes especially critical for historic buildings, where precise calibration of material properties significantly impacts hygrothermal analysis outcomes^3^. Such complexity intensifies in high-altitude mountain environments due to rapid temperature fluctuations, distinct microclimates, and considerable exposure to snow and wind.

A notable challenge in mountain contexts is the limited availability and reliability of climate data, particularly solar radiation models, due to complex terrain and scarce high-resolution monitoring^4,5^. The existing literature primarily examines residential buildings at high altitudes, highlighting passive strategies like airtightness, improved insulation, sunspaces, and radiant barriers to enhance thermal comfort and energy efficiency^6–11^. Studies on future climatic scenarios further emphasize the risks associated with overheating in retrofitted historic alpine buildings lacking active cooling systems^12,13^, reinforcing the importance of integrated, resilient retrofit strategies. The literature also underlines the critical role of occupant adaptation and passive comfort solutions in extreme climates^14^, alongside comprehensive life-cycle environmental evaluations for high-altitude structures^15^.

However, detailed hygrothermal analyses for historic mountain refuges and alpine structures dedicated to tourism, dining, or as actual shelters remain rare, and accessible datasets for these contexts are particularly lacking.

This Data Descriptor presents a multi-year (2020–2025) monitoring dataset from Rifugio Boè (2,871 m, Dolomites). The dataset comprises: (i) meteorological measurements on the south-east façade (136°) air temperature, relative humidity, global tilted irradiance, and driving rain; (ii) indoor air temperature and relative humidity in six rooms with different uses; and (iii) temperature and relative humidity at three depths within the retrofitted south-east wall (in-wall, behind-insulation, on-insulation). All channels are time-synchronised, quality-flagged, and distributed as CSV together with sensor metadata (make/model, accuracy, units, location and depth).

The dataset was created to support hygrothermal studies in high-altitude historic buildings, where boundary conditions and material behavior are difficult to characterize. Potential reuse includes deriving realistic boundary conditions, calibrating and validating hygrothermal models, assessing the durability of internal insulation in extreme climates, and benchmarking moisture-risk indicators.

## Methods

### Monitoring system installation and experimental design

**Fig. 1:**
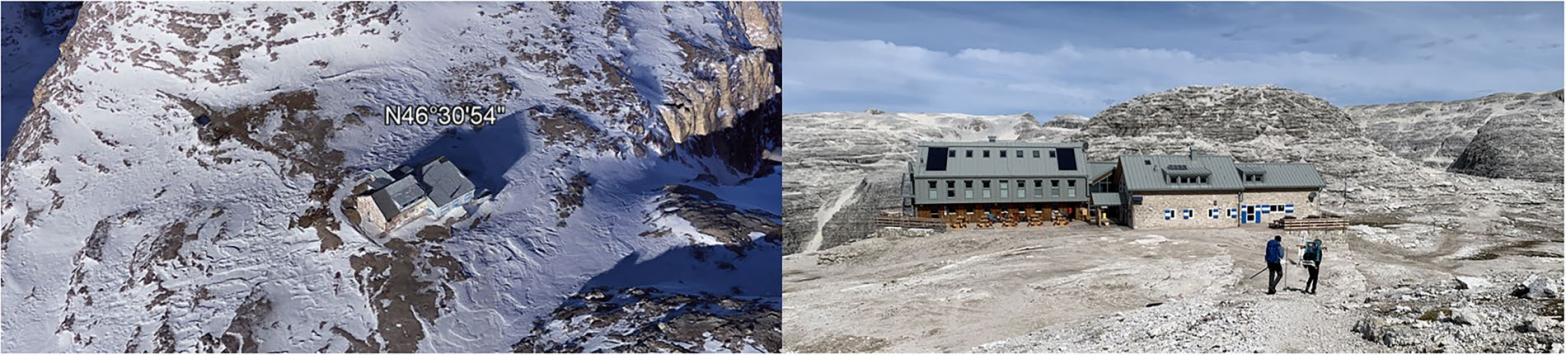
(left) Google Earth Pro aerial view of Rifugio Boé, captured from above, illustrating its location and setting in the alpine terrain. (right) Photograph taken during a site visit, showing the south-east façade and surrounding landscape.

During the 2020 renovation of Rifugio Boé (2871 m a.s.l.) in the Dolomites, a detailed monitoring system was established to capture essential climatic and hygrothermal data. The objective was to accurately and continuously record external and internal environmental conditions, including in-wall measurements across the stratigraphy, providing comprehensive data on an alpine building at high altitude (Fig. 1).

**Fig. 2:**
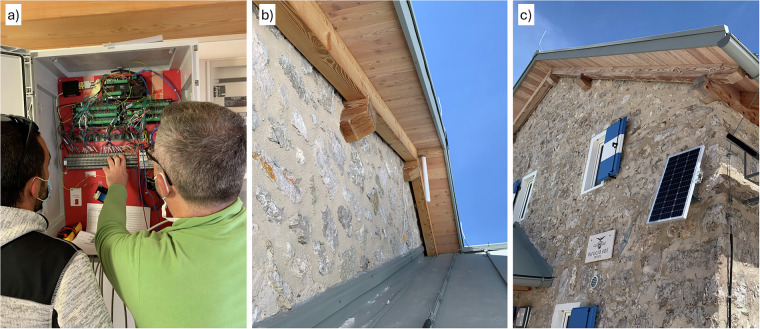
(**a**) Monitoring box containing the CR6 data logger, multiplexer, power supply, modem, sensor connections, and cabling, secured in a lockable enclosure. (**b**) Directional antenna mounted on the façade, connected to the modem with a low-loss cable to ensure data transmission. (**c**) Photovoltaic panel providing autonomous power supply.

A Campbell Scientific CR6 data logger, paired with an AM16/32B multiplexer, was selected for reliability, energy efficiency, and capability to manage multiple sensors Fig. 2(a). Data storage occurs locally in internal memory and on a robust 2 GB microSD card, ensuring data preservation even during extended communication outages. Data is transmitted hourly via a 3 G modem (Telit HT910E) utilizing an M2M SIM card and connected to a high gain omnipolar antenna, enhancing signal strength Fig. 2(b). A Dynamic DNS service ensures continuous remote access despite dynamic IP addresses.

Due to the refuge’s seasonal operation and lack of electrical power during winter, an autonomous photovoltaic power system was implemented. This comprises a 12 V / 44 Ah AGM battery, a 60 Wp photovoltaic panel oriented southwest, and a 5 A charge regulator. Energy-optimized software minimizes modem operation to conserve energy, enabling independent system operation for up to two weeks without sufficient solar charging Fig. 2(c).

## Data Record

This section provides a concise description of the monitoring layout, sensor types, and mounting positions/heights. All sensors used, along with their specific characteristics, are listed in Table 2.

### External environmental monitoring

**Fig. 3:**
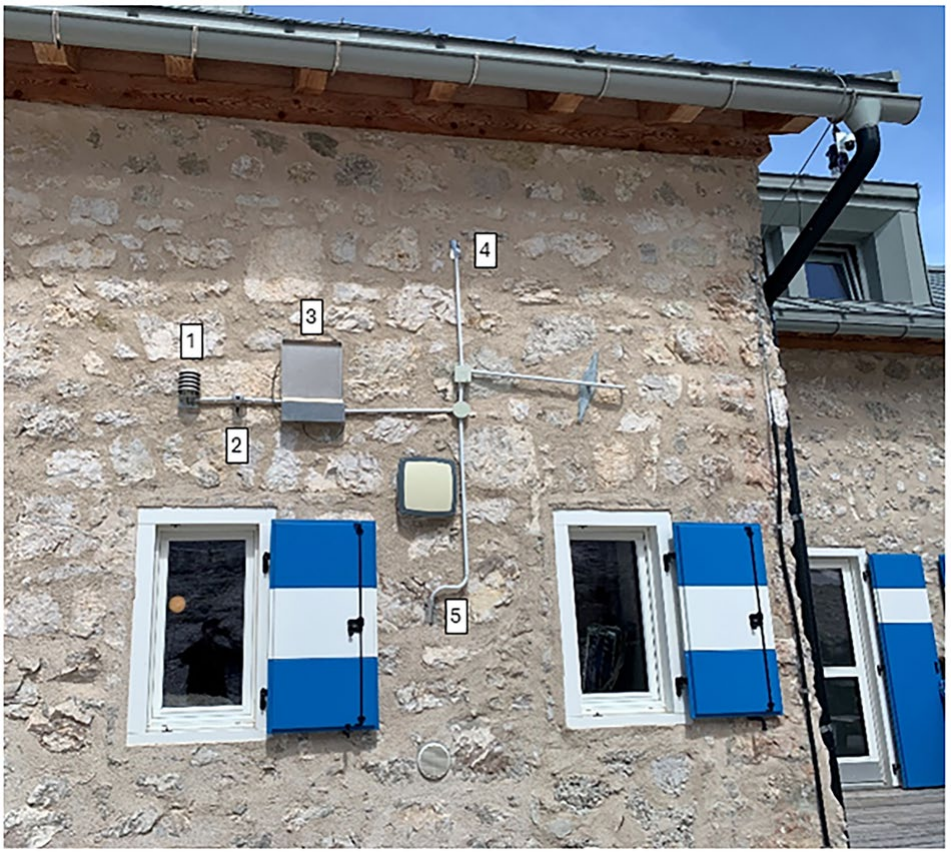
Installation on the historic wing showing the façade-mounted instruments: (1) a shielded air temperature/relative humidity sensor, (2) a Hukseflux SR05 pyranometer, (3) an SMT Research RG-01 driving-rain gauge, (4–5) and two Pt100 wall-surface temperature probes at the mid-heights of the ground and first floors. The Pt100 sensors were adhered to the wall surface using thermally conductive paste and covered with the same finishing coating as the surrounding area to ensure representative temperature measurements.

Sensors are installed on the south-east façade (136°) at approximately 2 m above ground level and are visible in Fig. 3. The pitched roof with exposed gutter provides intermittent shading from solar radiation and partial shelter from driving rain. (1) air temperature and relative humidity are measured with an Amphenol Telaire T9602 fitted with a radiation shield; (2) global tilted irradiance with a Hukseflux SR05 pyranometer; and (3) driving rain with an SMT Research RG-01 gauge; (4–5) surface temperature is measured by two Pt100 sensors mounted so that their elevations correspond to the mid-height of the ground floor and the mid-height of the first floor.

**Fig. 4:**
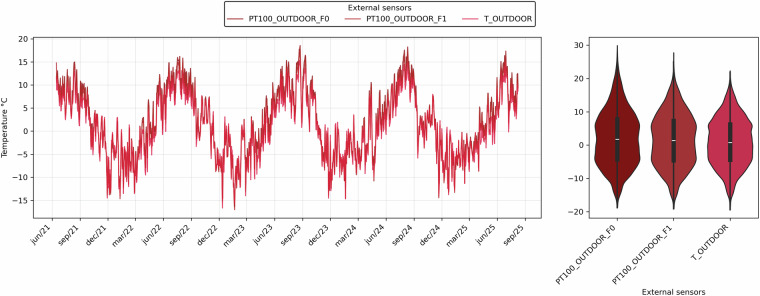
Hourly temperature values from two surface sensors and the external air temperature sensor, with violin plots of daily averages highlighting differences among the three sensors.

**Fig. 5:**
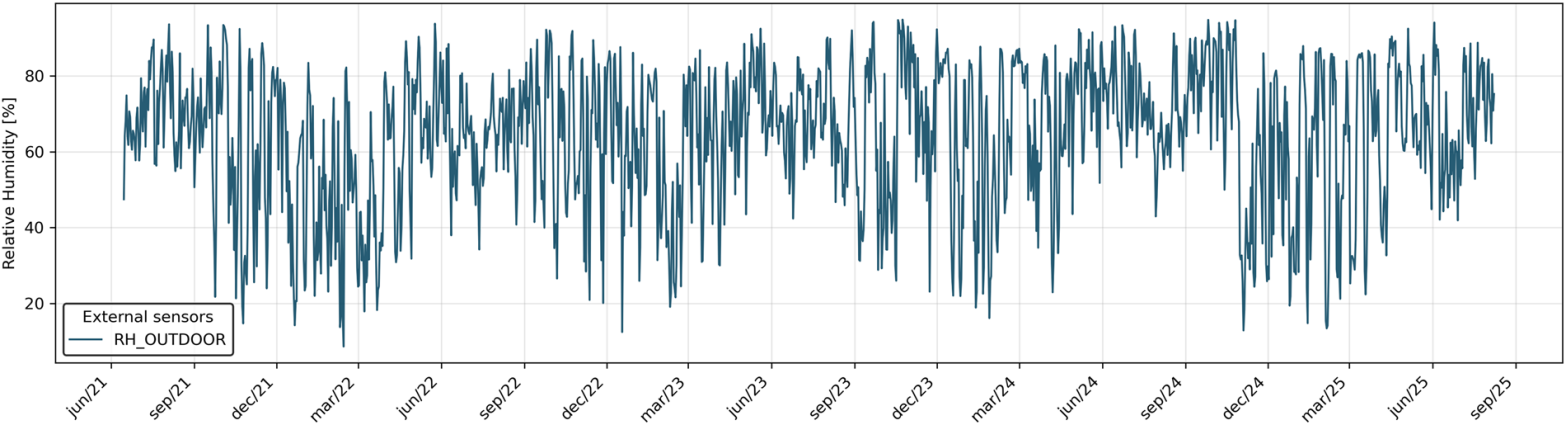
Hourly averaged relative humidity values recorded by the external sensor.

All sensors connected to the CR6 data logger recorded data every 5 minutes, which were then aggregated to hourly values using a Python script. Ambient and surface temperatures, as well as ambient relative humidity, were processed as hourly averages. Figure 4 illustrates these temperature data, showing the hourly values of the two surface sensors and the external air temperature, together with a violin plot of daily averages to highlight the differences among the three sensors. Figure 5 shows the relative humidity values recorded by the external sensor over the entire monitoring period, averaged on an hourly basis.

**Fig. 6:**
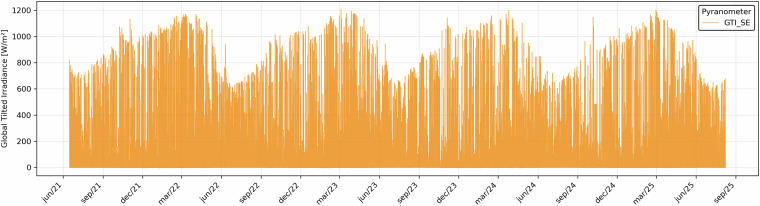
Hourly average solar radiation values recorded by the pyranometer.

Solar radiation values measured by the pyranometer were also aggregated as hourly averages. Figure 6 presents the resulting hourly average values of solar radiation.

**Fig. 7:**
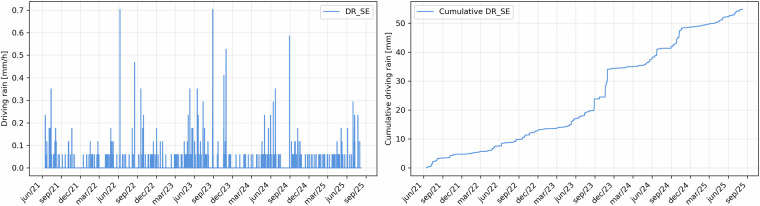
Driving rain: hourly sums in mm/h (left) and cumulative values over time (right).

Rainfall, recorded by a tipping-bucket rain gauge, was aggregated as hourly sums. Since the instrument measures the number of tips, the raw data were converted into precipitation depth (mm/h) using the calibration factor from the datasheet. Each tip corresponds to a water volume of 5.44 cm³ over a collecting area of 929 cm², equivalent to 0.05856 mm per tip. The hourly rainfall intensity was therefore calculated as

$${{\rm{P}}}_{1{\rm{h}}}={{\rm{N}}}_{{\rm{tips}}}\times 0.05856[{\rm{mm}}/{\rm{h}}]$$

where N_tips_ is the number of tips per hour. Figure 7 shows both the hourly rainfall intensities and the cumulative values over the monitored period. It should be noted that these data represent driving rain on a vertical surface and cannot be directly compared with standard precipitation measurements on a horizontal plane. Furthermore, the presence of the roof overhang reduces the actual amount of rain reaching the monitored wall compared to an unprotected surface. In addition, the rain gauge was positioned at a height that, in theory, should prevent it from being covered by snow. However, photographs taken during winter show that snow accumulation occasionally reached this level. Furthermore, temperatures well below 0 °C could freeze the collected water, blocking the tipping mechanism and reducing the number of recorded tips. It is therefore difficult to define what a “realistic” value might be under such extreme conditions. For this study, conventional values are reported, but readers are encouraged to consider these limitations.

### Internal environmental monitoring

Internal conditions were monitored with self-contained, battery-powered Lascar EL-USB-2 sensors. These sensors recorded air temperature and relative humidity, which were aggregated into hourly averages. Batteries were replaced and data downloaded annually during the summer access period. Despite this maintenance, occasional short interruptions occurred, resulting in limited data loss.

The stand-alone loggers were installed in the historic building and grouped by floor:

**Ground floor (GF):**

- two loggers in the Dining Room (GF_DiningRoom_A and GF_DiningRoom_B),
- one in the GF_MultipurposeRoom,
- one in the GF_Bathroom,
- and one in the GF_DryingRooms.

**First floor (FF):**

- two loggers in the guest room (FF_GuestRoom_A and FF_GuestRoom_B),
- one in the FF_NorthBedroom,
- and one in the south-facing FF_Bathroom_South.

**Fig. 8:**
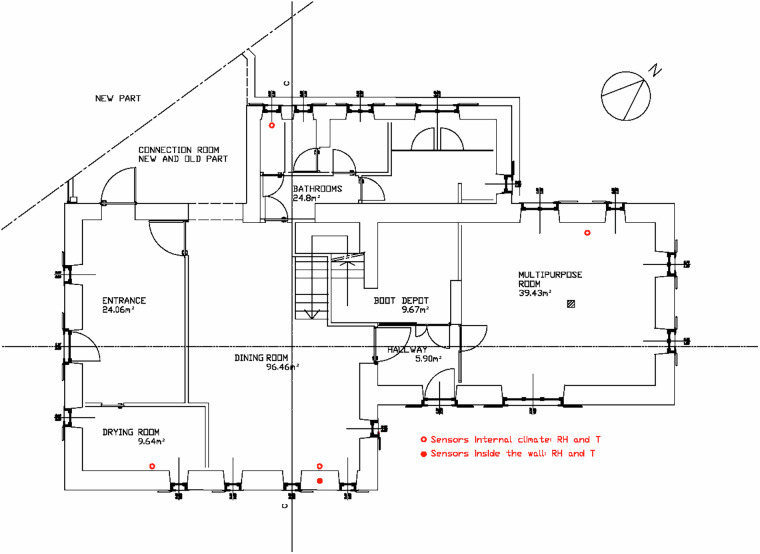
Ground Floor deployment of stand-alone indoor loggers (○: T, RH) and in-wall probes (●: T, RH). Locations: GF_DiningRoom (A and B), GF_MultipurposeRoom, GF_Bathroom, and DryingRooms. The dashed area indicates the connection to the new building.

**Fig. 9:**
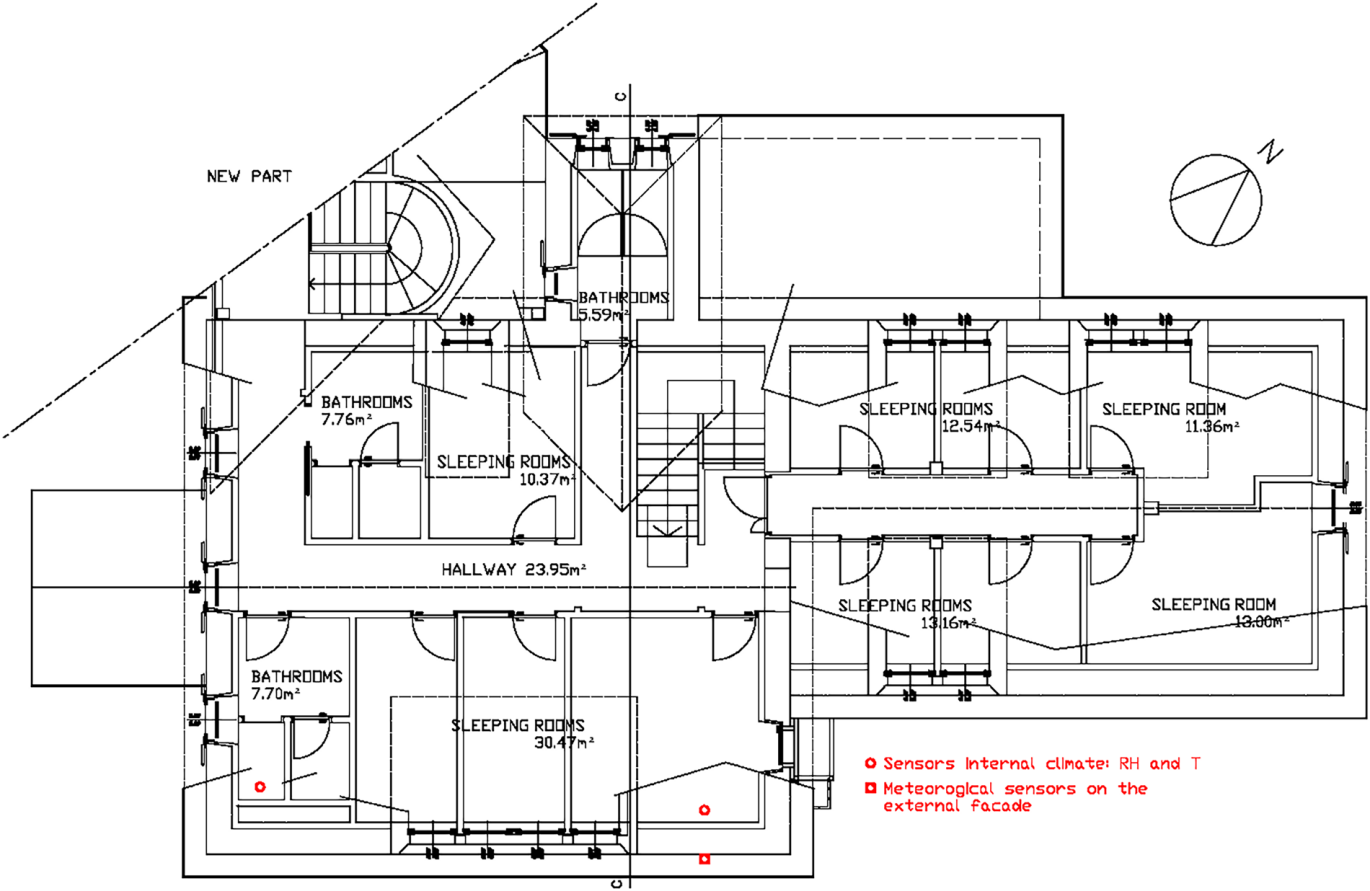
First Floor deployment of stand-alone indoor loggers (○: T, RH) and façade-mounted meteorological array (■) on the south-east wall (136°). Locations: FF_GuestRoom, FF_NorthBedroom, FF_Bathroom_South.

This deployment sampled different orientations and patterns of use. Sensor locations are shown in Fig. 8 (ground floor) and Fig. 9 (first floor).

**Fig. 10:**
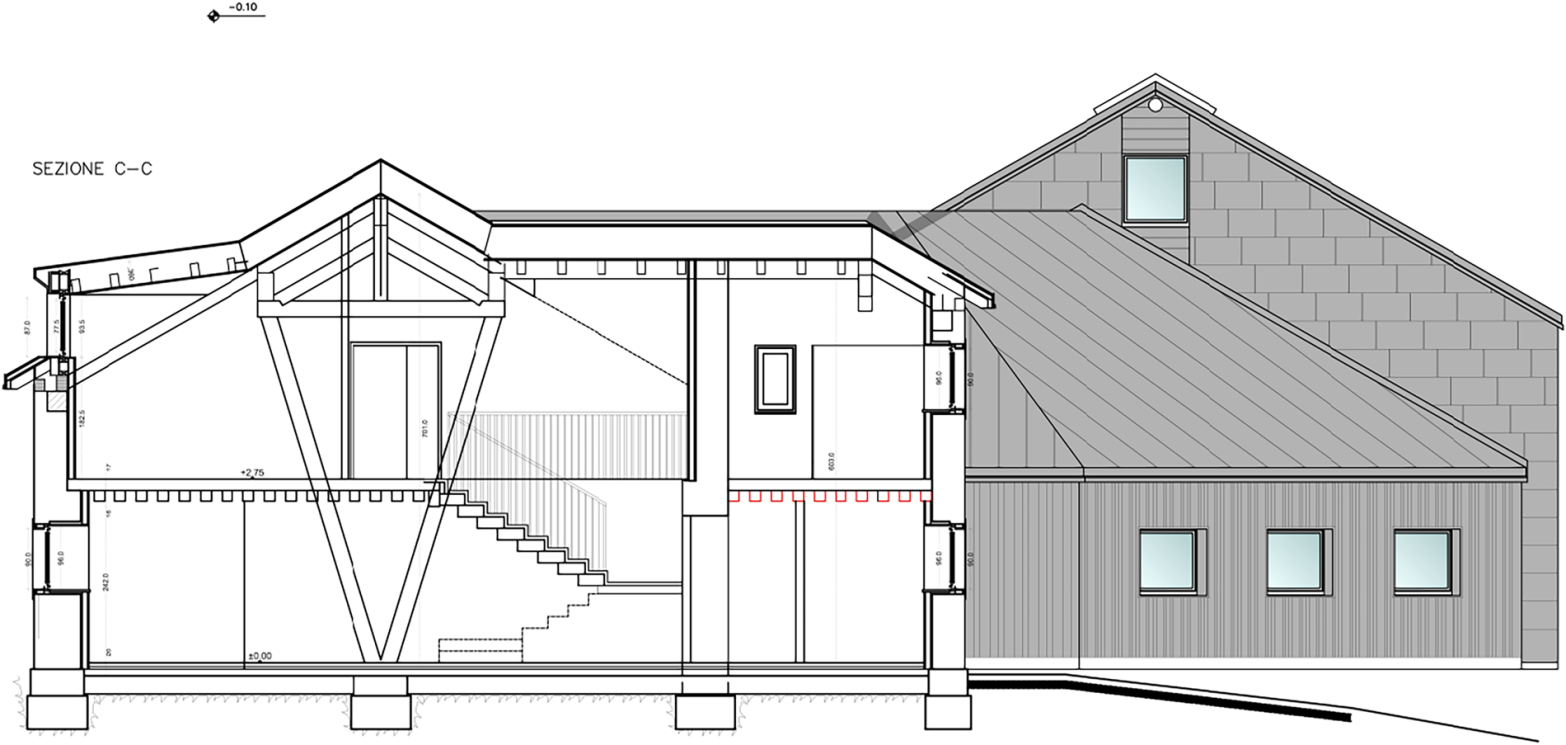
Cross section of the main building showing floor heights and roof overhang. The drawing highlights the internal spatial configuration and vertical distribution of the rooms discussed in Figs. 8, 9.

The drying room functions as a dedicated drying area; the dining room is seldom used, as meals are typically served outdoors or in the newer annex; the multipurpose room is often kept closed; and the bathrooms are used by visitors. To minimize visual impact, two loggers were installed in the dining room at different heights: one a few centimetres above the floor and another close to the ceiling. A similar low/high arrangement was adopted upstairs: in the guest bedroom, one sensor was concealed under a bed while the second was fixed near the ceiling on a wooden beam. The first-floor bathroom was usually locked and opened only for battery replacement. The north-facing bedroom was included to represent the most shaded exposure Fig. 10.

**Fig. 11:**
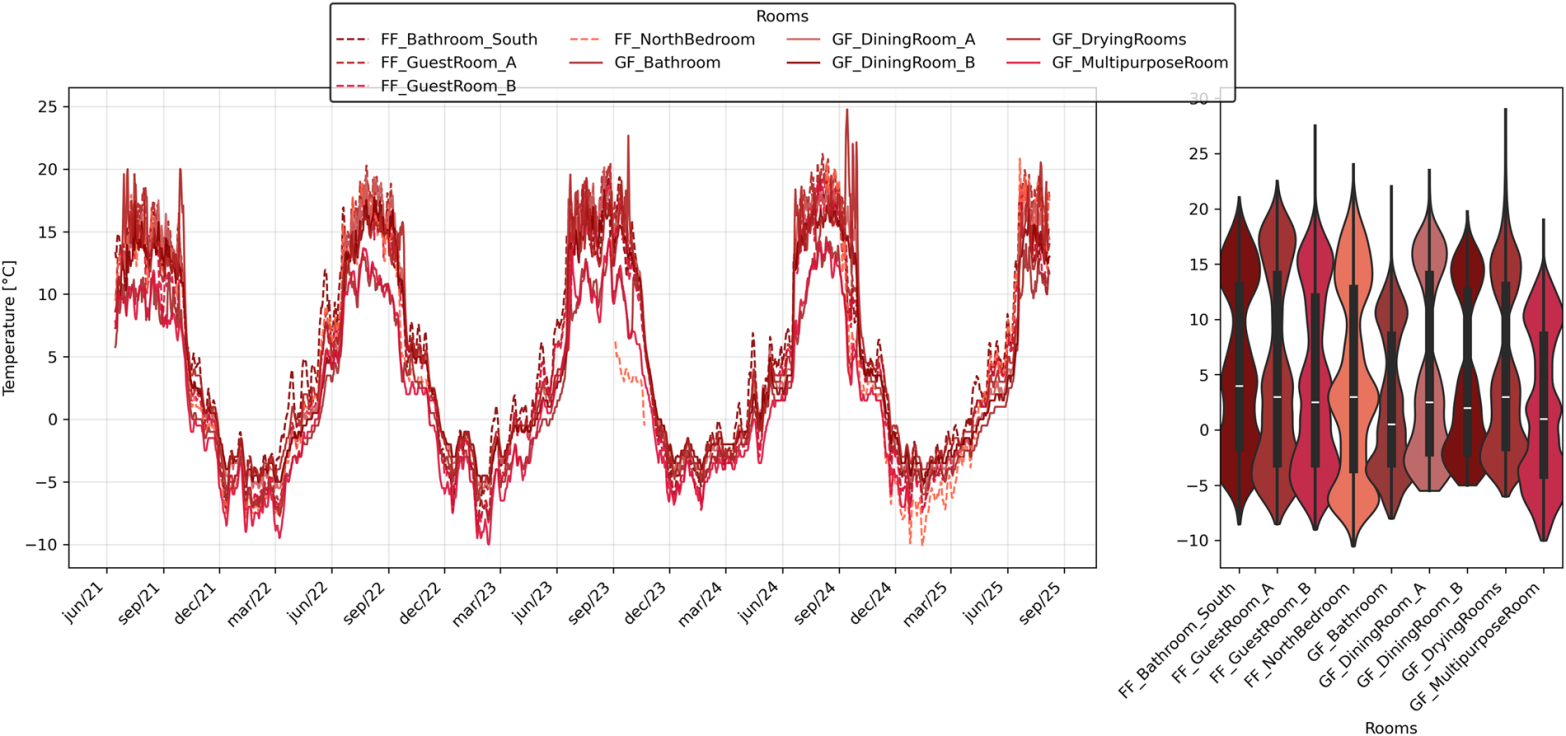
Daily mean temperature values for each room (left) and hourly distribution of temperature values shown by violin plots (right). Colours in red scale, dashed lines = FF (first floor), continuous lines = GF (ground floor).

**Fig. 12:**
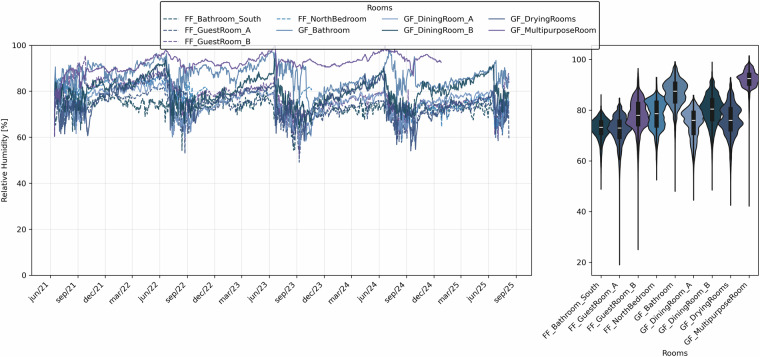
Daily mean relative humidity values for each room (left) and hourly distribution of relative humidity shown by violin plots (right). Colours in blue scale, dashed lines = FF (first floor), continuous lines = GF (ground floor).

Figures 11, 12 show the monitored temperature and relative humidity values, expressed as daily mean values for each room. The line plots represent the daily averages, while the violin plots display the distributions based on hourly data. Solid lines correspond to ground floor (GF) rooms, and dashed lines to first floor (FF) rooms. These visualizations provide a clear overview of the indoor environmental conditions, highlighting which rooms are more exposed to colder climates or higher humidity levels, and showing how the opening of the refuge during summer affects the indoor environment.

### Cross-sectional wall monitoring

During the 2020 retrofit, in-wall monitoring was implemented to capture hygrothermal conditions within the stratigraphy. Amphenol Telaire T9602 sensors were embedded at three depths in the south-east wall (136°):

(i) **in-masonry** (~20 cm from the interior surface),
(ii) **behind-insulation** (adhesive layer at the masonry–insulation interface),
(iii) **on-insulation** (beneath the internal plaster).

**Fig. 13:**
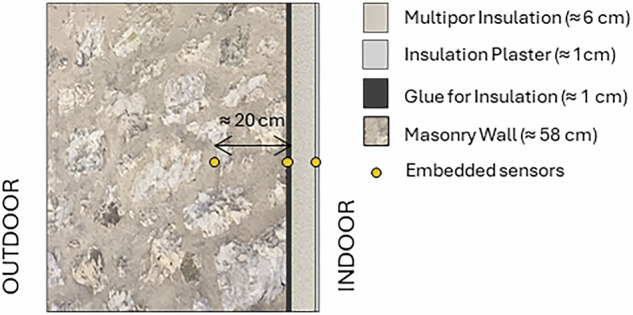
Monitored wall stratigraphy showing the arrangement of the different layers and the position of the embedded sensors across the masonry section.

Figure 13 shows the monitored stratigraphy with the position where the sensors are placed.

The monitored wall is representative of the envelope construction adopted throughout the renovated refuge. It consists of the original stone masonry, an internal insulation layer, and a lime-based plaster finish. Openings are fitted with triple-glazed windows featuring aluminium–wood frames, designed to ensure improved airtightness and thermal performance consistent with the retrofit objectives. The water absorption coefficient for the stone masonry was obtained from laboratory measurements, while the values for the other materials were derived from the manufacturers’ datasheets (see Table 1).

**Table 1:** Thermo-physical and hygric properties of the materials composing the monitored wall.

Layer/Material	Product name	Thickness [m]	Density [kg/m³]	Thermal Conductivity [W/m·K]	Water Vapour Resistance Factor [–]	Water Absorption [kg/m²·s⁰·⁵]
Stone masonry (mixture of Dolomite stone and historic mortar)	—	≈58 cm	—	—	—	0.0017 (lab measurements)
Adhesive mortar	Multipor Malta Leggera Collante	≈1 cm	350	0.12	15	0.2
Insulation	Multipor	≈6 cm	115	0.045	3	0.05
Indoor: Plaster	Kerakoll Geocalce Multiuso	≈1 cm	1300	0.54	13	0.4

The selected wall corresponds to the room originally targeted as the main dining area and was also used in preliminary pre-design simulations (see Fig. 9). Subsequent site inspections, however, revealed that this space is rarely used, as the refuge managers usually prefer serving meals in the newer annex or outdoor areas.

At each depth, three sensors were installed to provide redundancy, enable fault/outlier detection, and yield a more representative average signal.

**Fig. 14:**
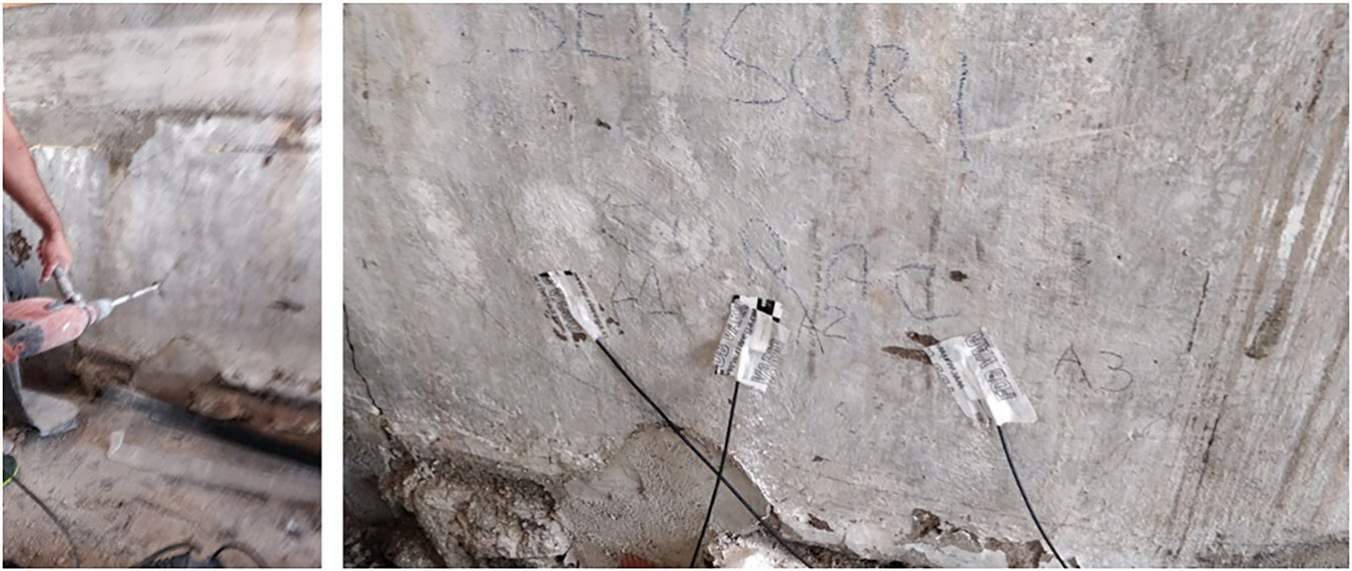
Drilling of the masonry to accommodate the sensors approximately 20 cm deep (left) and sensors placed in the holes (right).

Installation proceeded in phases. For the in-masonry set, boreholes were drilled to a depth of approximately 20 cm. Before placing each sensor at the bottom of its borehole, the sensing heads were protected with vapor-permeable covers (specifically, Riwega FDB EXT, S_d_ = 0.004 m). Once the sensors were positioned, the borehole openings were sealed with FLEXI BAND tape (Rothoblaas, S_d_ = 45 m, see Fig. 14).

**Fig. 15:**
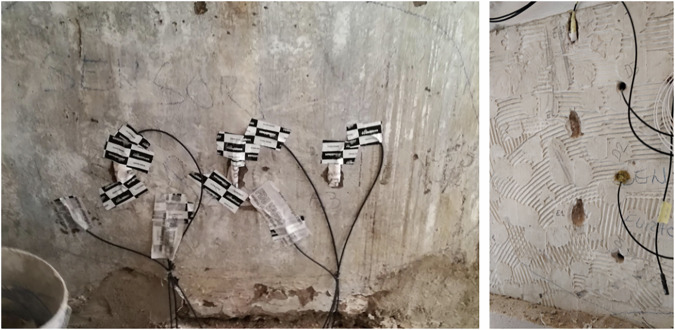
Images showing sensors placed inside the wall and those positioned in the layer beneath the insulation. A recess in the adhesive was left to accommodate and protect the covered sensors.

The behind-insulation set was embedded directly in the adhesive layer during the application process; the sensing heads were also protected with vapor-permeable covers (Riwega FDB EXT, S_d_ = 0.004 m), and a small recess was left in the mortar to safeguard the sensor body and wiring (see Fig. 15).

**Fig. 16:**
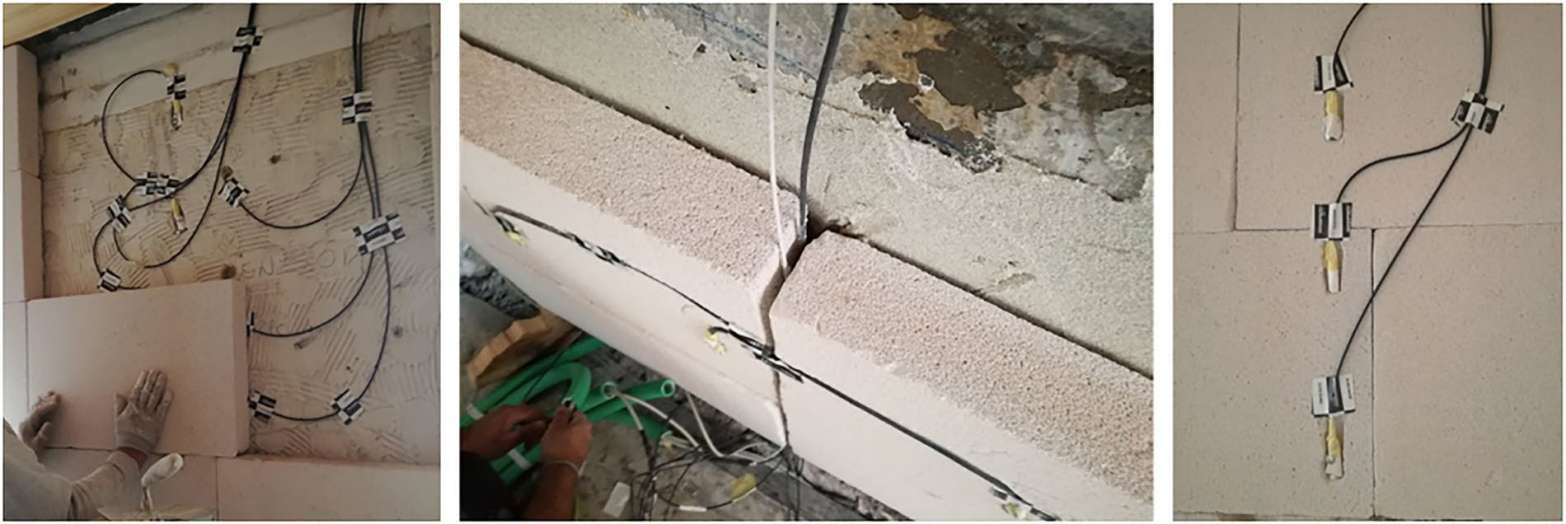
Installation of sensors on the insulation layer: sensors placed in the adhesive joints and covered by the insulating panels.

The on-insulation set was seated in shallow recesses cut into the insulation and subsequently covered by the finishing plaster. Cables were pre-routed and kept outside the measurement zones; sensor spacing and lead routing were arranged to minimize thermal/moisture disturbance. Details of the installation are illustrated in Fig. 16.

All sensors, except one, show coherent magnitudes and temporal evolution. In particular, the third probe embedded in the masonry layer (identified as *T03_WALL_F0* and *RH03_WALL_F0*) exhibited unrealistic values, most likely due to a connection failure or cable breakage that occurred during the monitoring period.

**Fig. 17:**
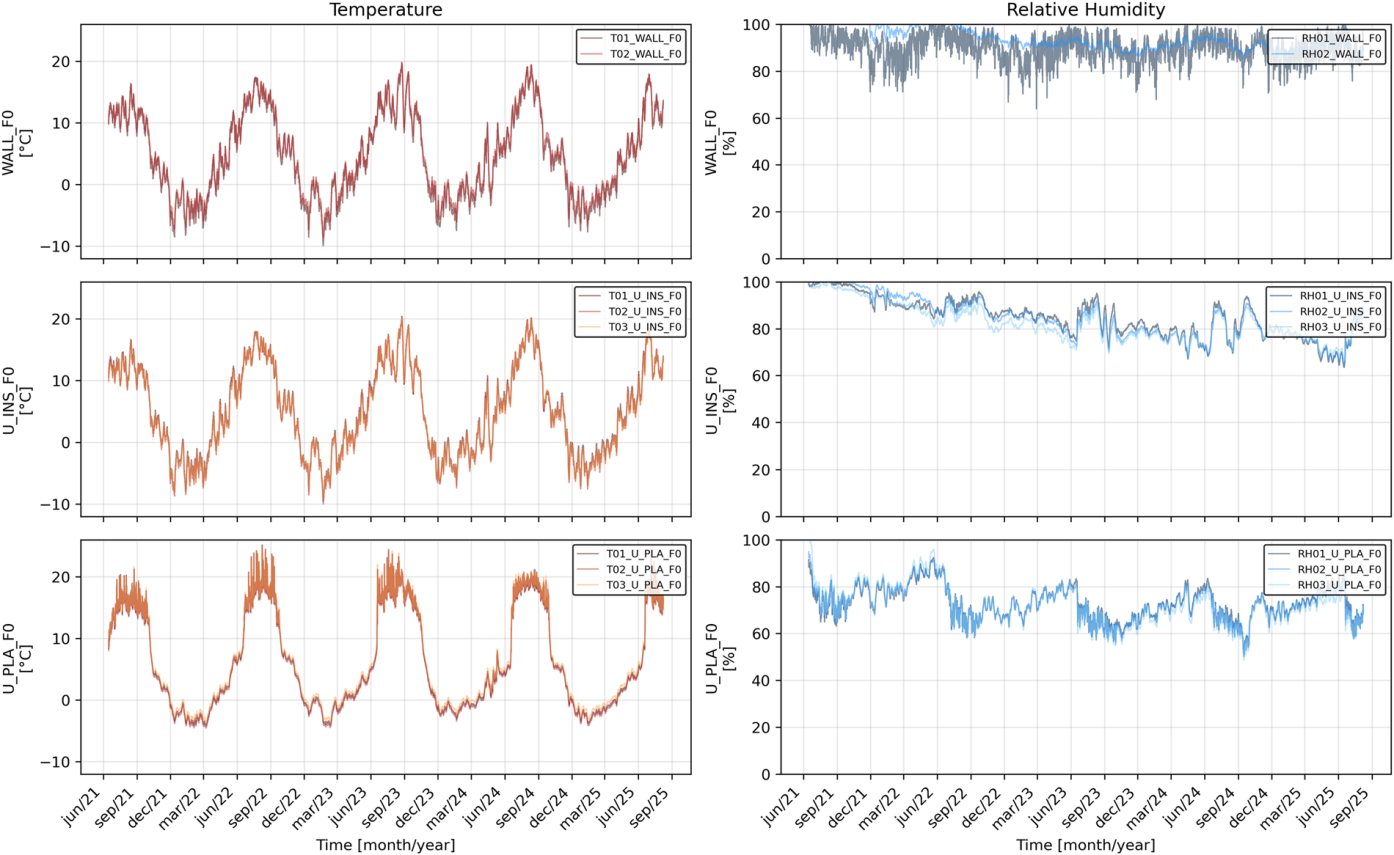
Temperature (left) and relative humidity (right) recorded by in-wall sensors installed at three depths within the monitored wall: in-masonry (*WALL_F0*), behind insulation (*U_INS_F0*), and on insulation (*U_PLA_F0*).

Consequently, this sensor was excluded from the averaged series, and only two sensors were retained at this position in the dataset. Figure 17 shows the temperature and relative humidity values recorded by each individual sensor. These plots illustrate the consistency among sensors installed at the same depth within the wall stratigraphy.

**Fig. 18:**
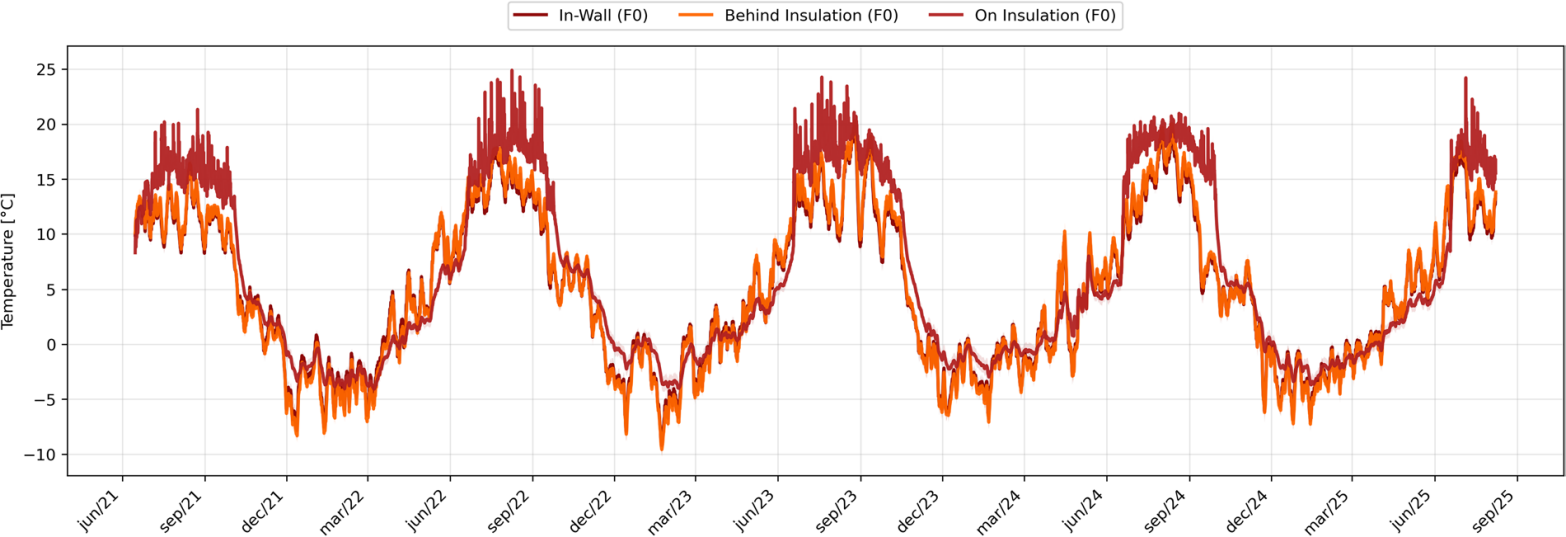
Hourly mean temperature values recorded at three depths of the monitored wall cross-section (in-masonry, behind insulation, and on insulation). Shaded bands indicate daily min–max ranges extended by the documented sensor uncertainty (±0.2 °C).

**Fig. 19:**
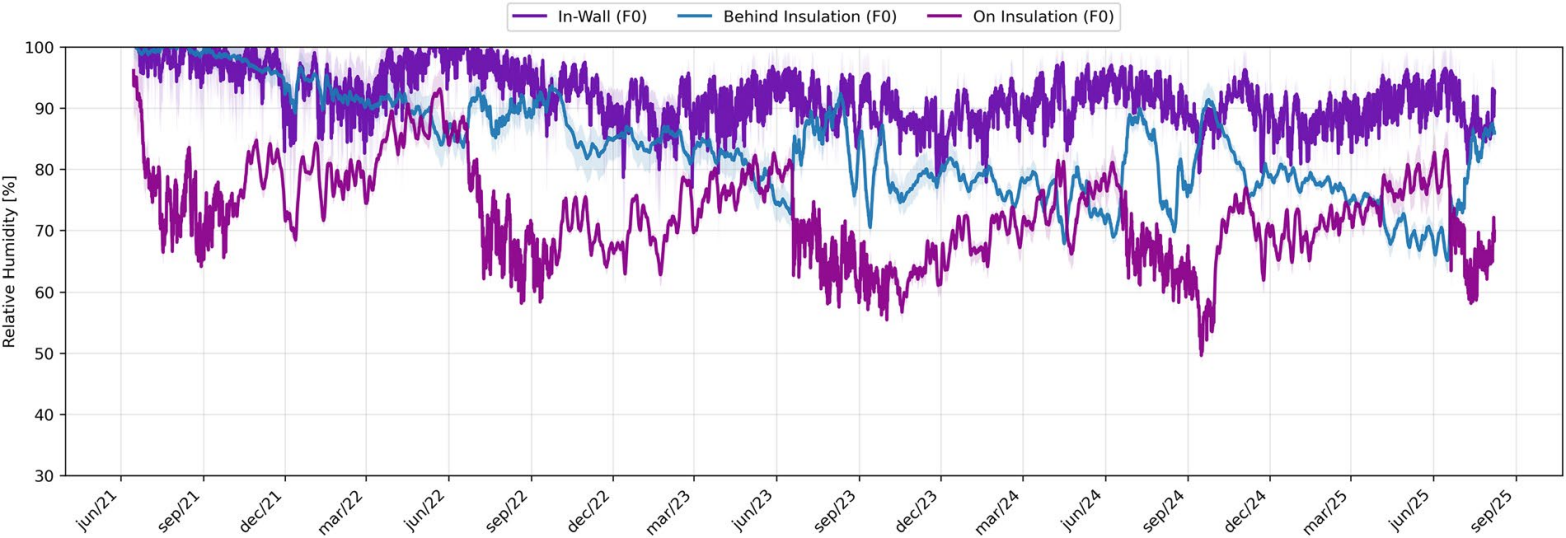
Hourly mean relative humidity values at the same three depths of the wall cross-section. Shaded bands show daily min–max ranges extended by the documented sensor uncertainty (±0.5%), limited to 0–100%.

In Figs. 18, 19, the mean temperature and relative humidity values for each position is shown. In these figures, the shaded transparent areas represent the measurement uncertainty arising from the sensor tolerance and variability among probes installed at the same depth. In the temperature plots (Fig. 18), these bands are extremely narrow, confirming the close agreement among the functioning sensors.

Table 2 lists full specifications (make/model, accuracy, units, channel IDs, orientation/coordinates or depth, logging interval, and quality-flag definitions).

**Table 2:** Sensor types used at Rifugio Boé, with measured variables, units, declared accuracy, power/placement, protection, and native logging interval. The SE façade is oriented 136°; in-wall sensors are at three depths (in-masonry ~20 cm, behind-insulation, on-insulation). GF/ FF denote ground/first floor.

Sensor type (make/model)	Variable(s)	Units	Accuracy (declared)	Power	Typical placement in this study	Protection	Logging interval
Amphenol Telaire T9602	Air temperature, Relative humidity	°C, %	± 0.5 °C; ± 2%RH	Logger-powered (wired)	Outdoor T/RH on SE façade (136°); in-wall at three depths (in-masonry ~20 cm, behind-insulation, on-insulation)	External: radiation shield. In-wall: vapor-permeable cover (Riwega FDB VARIO)	5 min
Lascar EL-USB-2 (stand-alone)	Air temperature, Relative humidity	°C, %	± 0.45 °C; ± 3%RH	Battery-powered (annual replacement)	Indoor rooms (Dining, Drying, Multipurpose, Bathrooms, Guest Room, North Bedroom)	—	60 min
Hukseflux SR05 (Second-Class pyranometer)	Global tilted irradiance (GTI)	W/m²	ISO 9060 Second Class; calibration uncertainty < 1.8% (k = 2); spectral range 285–3000 nm	Logger-powered (wired 5–30 VDC)	Mounted on SE façade (tilt = façade)	—	5 min
SMT Research RG-01 (driving-rain gauge)	Driving rain	mm/h (L/m²·h)	Resolution 0.059 mm per tip (5.44 cm³ over 929 cm² catchment); body ~30 × 43 × 6 cm; 12 m cable	Logger-powered (wired, Passive reed-switch)	SE façade, co-planar with wall	Partial roof/gutter shielding noted	5 min
Pt100 surface probes	Surface temperature	°C	± 0.1 °C	Logger-powered (wired)	SE façade wall surface at mid-GF and mid-FF elevations	—	5 min

## Data Record

### Repository and access

The dataset is hosted on Zenodo under 10.5281/zenodo.17473383^16^, release Version 4 (2025-11-10), licensed CC-BY-4.0.

### File formats and conventions

Primary files are CSV (UTF-8, comma-separated, dot decimal); Timestamps are ISO-8601 in timezone Europe/Rome (DST observed). Missing values are encoded as NaN. Units: air/surface temperature [°C], relative humidity [%], global tilted irradiance [W·m^−^²], driving rain [mm·h^−^¹].

### Variables and naming (Column Names)

#### External (SE façade, 136°)

- Meteorology: T_OUTDOOR, RH_OUTDOOR, GTI_SE, DR_SE
- Surface temperature (Pt100, two elevations): PT100_OUTDOOR_F0, PT100_OUTDOOR_F1

#### Indoor (stand-alone loggers)

**Ground Floor (GF):**

- GF_DiningRoom_A_Temp, GF_DiningRoom_A_Rh
- GF_DiningRoom_B_Temp, GF_DiningRoom_B_Rh
- GF_MultipurposeRoom_Temp, GF_MultipurposeRoom_Rh
- GF_Bathroom_Temp, GF_Bathroom_Rh
- GF_DryingRooms_Temp, GF_DryingRooms_Rh

**First Floor (FF):**

- FF_GuestRoom_Temp, FF_GuestRoom_Rh
- FF_NorthBedroom_Temp, FF_NorthBedroom_Rh
- FF_Bathroom_South_Temp, FF_Bathroom_South_Rh

**In-wall (SE wall, three depths)**

- Temperature: T01_WALL_F0, T02_WALL_F0, T03_WALL_F0 (in-masonry ~20 cm; behind-insulation; on-insulation)
- Relative humidity: RH01_WALL_F0, RH02_WALL_F0, RH03_WALL_F0
- Interface temperatures (behind insulation): T01_U_INS_F0, T02_U_INS_F0, T03_U_INS_F0
- Interface RH (behind insulation): RH01_U_INS_F0, RH02_U_INS_F0, RH03_U_INS_F0
- On-insulation temperatures (under plaster): T01_U_PLA_F0, T02_U_PLA_F0, T03_U_PLA_F0
- On-insulation RH (under plaster): RH01_U_PLA_F0, RH02_U_PLA_F0, RH03_U_PLA_F0
- Indoor surface temperatures (Pt100): PT100_INDOOR_F0, PT100_INDOOR_F1

A glossary of all variable names included in the dataset is provided in Table 3 to facilitate data reuse and interpretation.

**Table 3:** Glossary of variables contained in the dataset.

Column name	Variable / Description	Units
**TIMESTAMP**	Date and time of each measurement (ISO-8601, timezone Europe/Rome, DST observed).	—
**FF_GuestRoom_A_Temp, FF_GuestRoom_B_Temp**	Indoor air temperature in first-floor guest room, sensors A and B (two heights).	°C
**FF_GuestRoom_A_Rh, FF_GuestRoom_B_Rh**	Indoor relative humidity in first-floor guest room, sensors A and B.	%
**GF_DiningRoom_A_Temp, GF_DiningRoom_B_Temp**	Indoor air temperature in ground-floor dining room, sensors A and B (two heights).	°C
**GF_DiningRoom_A_Rh, GF_DiningRoom_B_Rh**	Indoor relative humidity in ground-floor dining room, sensors A and B.	%
**FF_Bathroom_South_Temp, FF_Bathroom_South_Rh**	Indoor air temperature and relative humidity in first-floor south-facing bathroom.	°C, %
**GF_DryingRooms_Temp, GF_DryingRooms_Rh**	Air temperature and relative humidity in the ground-floor drying room.	°C, %
**FF_NorthBedroom_Temp, FF_NorthBedroom_Rh**	Indoor air temperature and relative humidity in the north-facing first-floor bedroom.	°C, %
**GF_Bathroom_Temp, GF_Bathroom_Rh**	Indoor air temperature and relative humidity in the ground-floor bathroom.	°C, %
**GF_MultipurposeRoom_Temp, GF_MultipurposeRoom_Rh**	Indoor air temperature and relative humidity in the ground-floor multipurpose room.	°C, %
**T01_WALL_F0, T02_WALL_F0, T03_WALL_F0**	Temperature inside masonry (approx. 20 cm from interior surface), three replicated sensors for redundancy.	°C
**T01_U_INS_F0, T02_U_INS_F0, T03_U_INS_F0**	Temperature at the interface between masonry and insulation layer (“behind insulation”).	°C
**T01_U_PLA_F0, T02_U_PLA_F0, T03_U_PLA_F0**	Temperature on the insulation surface, just beneath the internal plaster (“on insulation”).	°C
**RH01_WALL_F0, RH02_WALL_F0, RH03_WALL_F0**	Relative humidity inside masonry (approx. 20 cm from interior surface), three replicated sensors.	%
**RH01_U_INS_F0, RH02_U_INS_F0, RH03_U_INS_F0**	Relative humidity at the masonry–insulation interface (“behind insulation”).	%
**RH01_U_PLA_F0, RH02_U_PLA_F0, RH03_U_PLA_F0**	Relative humidity on the insulation surface beneath the plaster (“on insulation”).	%
**PT100_INDOOR_F0, PT100_INDOOR_F1**	Surface temperature probes on indoor wall surfaces at ground and first floor.	°C
**PT100_OUTDOOR_F0, PT100_OUTDOOR_F1**	Surface temperature probes on outdoor wall surfaces at ground and first floor.	°C
**T_OUTDOOR**	Outdoor air temperature measured on the SE façade (136°).	°C
**RH_OUTDOOR**	Outdoor relative humidity measured on the SE façade (136°).	%
**GTI_SE**	Global tilted irradiance on the SE façade (solar radiation incident on wall plane).	W·m^−^²
**DR_SE**	Driving rain intensity measured on the SE façade (rain impacting vertically on wall surface).	mm·h^−^¹

## Technical Validation

Overall, the measurements behave as expected: outdoor series follow weather variability, indoor loggers respond plausibly to occupancy and ventilation, irradiance approaches zero at night, and driving-rain totals align with storm periods (check done with close weather stations). The in-wall temperature and humidity probes were embedded in the stratigraphy but are probably not reliable. Within triplets at the same depth some sensors diverge with drifts, offsets, or step changes, likely due to local material heterogeneity, imperfect sealing of the borehole, or occasional sensor malfunction. In-wall readings should therefore be treated as indicative for trends and relative changes, while indoor, outdoor, and surface measurements should be used for precise quantitative analysis.

### Summary statistics

Time span: 2020-09-23 → 2025-08-08 (hourly, timezone-aware Etc/GMT-1). Dataset size: 42,733 hourly records × 44 columns.

Sampling grid:

**Table:** 

Item	Value
Time span	2020-09-23 to 2025-08-08 (Europe/Rome)
Interval	1 hour (regular grid)
Rows × Columns	42,733 × 44
Exact 1-h steps (with ± 1 s tolerance)	100%

A data completeness analysis was carried out to quantify the proportion and duration of missing records for each variable. The dataset shows an excellent level of completeness, with most channels exhibiting less than 0.05% missing values. Short interruptions, typically lasting only a few hours, occurred occasionally during routine maintenance or temporary communication failures.

The analysis considered the actual activation date of each sensor to avoid bias from pre-installation periods. Indoor standalone loggers show slightly higher data gaps due to their battery-powered operation and annual data download, while all other sensors maintained stable continuity throughout the monitoring period. The longest single gaps are shorter than 12 hours.

A detailed summary of the completeness metrics has been added to the Zenodo repository and can also be generated directly by running the provided “ZENODO_Print.py” script.

## Data Availability

The full dataset is available from Zenodo at 10.5281/zenodo.17473383^16^, released under a CC-BY-4.0 licence. The repository includes all raw data files, processed CSV datasets, a readme file and the associated Python scripts. Previous Zenodo records correspond to earlier versions automatically generated by Zenodo during updates to the repository. The latest release consolidates both the data and code and should be considered the primary reference for reuse and citation.

## Code availability

Data pre-processing and aggregation were carried out using Python scripts specifically developed for this study. These scripts are openly accessible on Zenodo together with the dataset at 10.5281/zenodo.17473383^16^.
